# Health Insurance Coverage of Recommended Gender-Affirming Health Care Services for Transgender Youth: Shopping Online for Coverage Information

**DOI:** 10.1089/trgh.2018.0055

**Published:** 2019-04-11

**Authors:** Nadia L. Dowshen, Julie Christensen, Siobhan M. Gruschow

**Affiliations:** ^1^ Department of Pediatrics, Children's Hospital of Philadelphia, Philadelphia, Pennsylvania.; ^2^ Sidney Kimmel Medical College, Thomas Jefferson University, Philadelphia, Pennsylvania.

**Keywords:** health policy, insurance coverage, transgender health, transgender youth

## Abstract

We assessed online health insurance plan indication of coverage and accessibility of information for recommended services for transgender youth (TY). Content analysis was performed for plans used at a pediatric Gender Clinic by reviewing information about coverage of puberty blockers, hormones, masculinizing chest surgery, and counseling. Transgender-specific exclusions and the time required for the research assistant to review each plan's online information were noted. No plan (0%; *n*=36) indicated coverage of all four categories of recommended services online. Forty-nine percent indicated ≥1 transgender-specific exclusion. The median time required for a research assistant to review online coverage information for each insurance plan was 50 min. Efforts are needed to ensure that online insurance information is accessible and updated in accordance with policy and coverage recommendations for TY.

## Introduction

An estimated 1% of youth in the United States identify as transgender,^1,2^ meaning that their gender identity differs from their sex assigned at birth. Transgender and gender nonconforming (GNC) youth have unique and specific health care needs. Due to the stigma and discrimination experienced by GNC youth, rates of medical and mental health challenges are high, including depression, anxiety, suicide attempts, eating disorders, substance abuse, and STIs.^3–5^ Among young transwomen, HIV infection rates are as high as 25%.^3,6^

As a result of the specific health needs of transgender youth (TY) and based on their developmental stage, the World Professional Association of Transgender Health and the Endocrine Society have made treatment recommendations for transgender adolescents that in many cases may differ from adults. Medically necessary services for the treatment of gender dysphoria in children and adolescents may include the following: (1) puberty blockers that are not typically recommended for adults, (2) gender-affirming hormone therapy that is recommended generally in mid-to-late adolescence, (3) masculinizing chest surgery (other gender-affirming surgeries are not typically recommended until after age 18), and (4) behavioral health counseling that may not be necessary in adults seeking care in a medical consent model, but is a critical component of the pediatric transgender health care model.^7–9^

With these unique medical needs, health insurance coverage is critical to ensure that transgender and GNC youth receive services recommended by their health care providers to promote their health and well-being.

Despite advances in clinical models of care and therapeutics, transgender and GNC youth face many barriers to accessing quality health care. According to the American College of Physicians in 2015, transgender people were more likely to be uninsured compared with either the general population or cisgender lesbian, gay, and bisexual persons.^10^ Furthermore, even when transgender people obtain public or private insurance, their plans may have severely limited coverage for medically necessary transition-related services and procedures.^11–13^ One in four adult (25%) respondents to the 2015 U.S. Transgender Survey reported a problem in the past year with their insurance, such as being denied coverage of transition-related care or denied routine care because they were transgender.^14^

Further such barriers to receiving care can ultimately have a negative impact on the health and well-being of transgender patients and their families.^15^ Less is known, however, about how the insurance landscape impacts access to medically necessary services for transgender and GNC youth, but early qualitative work suggests that insurance issues create major barriers for these youth and their families.^16^

Recent health insurance policy changes have impacted the coverage landscape for transition-related care and may increase access to recommended services. One such transgender-inclusive policy is Section 1557 of the Affordable Care Act (ACA), signed into law in May 2016, which prohibits publicly funded insurers from discriminating on the basis of sex and gender identity.^17^ The policy does not require providers or plans to specifically cover transition-related services. However, those who do not remove blanket denial policies and transgender-specific exclusions or who refuse to cover a service for a transgender patient who is covered to treat other conditions will be in violation of the rule.^18^

Given that the policy does not mandate coverage of medically necessary transition-related services, transgender and GNC youth and their families are forced to shop around for insurance policies that offer the coverage they need.^19^ Yet, even highly educated adolescents and young adults, regardless of gender identity, find the process of selecting and enrolling in health insurance to be confusing and challenging.^20^ Minimal data exist regarding the accessibility of online insurance coverage information relevant to transgender and GNC youth. The objective of this study was to assess the online accessibility and indication of coverage of recommended medical and mental health services for TY by major payors serving a large U.S. metropolitan area, pediatric hospital-based, multidisciplinary gender clinic.

## Methods

### Data collection

We conducted an online content analysis of publicly available insurance plan coverage information related to gender-affirming care. The study surveyed the most frequent insurance payors to Children's Hospital of Philadelphia, serving patients located primarily in Pennsylvania. Content analysis was performed by five student research assistants from July through August 2016. We developed a protocol for reviewing the websites to simulate the consumer perspective on accessing coverage information, similar to mystery shopping methodology, which has been used previously to uncover specific barriers to accessing sexual and reproductive health care.^21,22^ Data were collected through Survey Monkey, an online cloud-based survey development software (SurveyMonkey, Inc., San Mateo, CA).

Two study team members separately reviewed each of the insurance providers' websites using the following protocol: (1) review insurer website for policies regarding coverage of transition-related services (i.e., puberty blocking medications, HRT, surgical procedures, and behavioral health counseling); (2) search for company policies surrounding gender and sex more generally (i.e., nondiscrimination policy, blanket exclusions, ACA Section 1557, etc.); (3) perform in-depth review of individual plan's Summary of Benefits and Coverage (SBC), member manual, provider manual, drug formulary, member rights and responsibilities, plan highlights, drug lists, exclusions, special bulletins and policies, and definitions of medically necessary treatment; and (4) use browser search function to search for all the following keywords in the SBC and any other relevant pages/documents: puberty blockers, GnRH agonists, transgender, gender transition, trans-sexual, sex reassignment surgery, sex change, gender reassignment surgery, cross-gender hormones, cross-sex hormones, hormone replacement therapy, gender identity, gender identity disorder, gender dysphoria, Supprelin, Vantas, Lupron, Spironolactone, feminizing, feminization, masculinizing, masculinization, prior authorization, and ICD10 codes F64, ICD9 codes 302.

Student research assistants discussed any discrepancies in their findings; if they were unable to resolve differences in coding, the study principal investigator was consulted. The protocol was exempt from review by the University of Pennsylvania Institutional Review Board.

### Measures

Adequacy of indication of gender-affirming care coverage was measured based on indication of reimbursement for the following four services that are recommended treatment for transgender children and adolescents: (1) puberty blockers, (2) gender-affirming hormone therapy, (3) masculinizing chest surgery, and (4) mental health counseling. Data were coded as covered, failure to cover, or unclear. Services were defined as “covered” if the plan explicitly stated coverage of the service for treatment of gender dysphoria. Those defined as “failure to cover” meant the plan explicitly stated that it did not cover services for treatment of gender dysphoria. Finally, plans were coded as “unclear” if research assistants were unable to determine whether services were covered or the language in the policy was not definitive.

Transgender-specific exclusions were measured in the following two ways: (1) we evaluated whether the plan explicitly stated denial of coverage for a recommended service because the service was related to treatment of gender dysphoria or gender transition, and (2) whether the plan denied coverage based on a patient's gender marker; that is, denial of pap smear for a transgender male based on a male gender marker.

Accessibility of plan information was measured by tracking the time required for active review by medical student research assistants. Review times were recorded at 10-min intervals from 0 to 60 min with the final indicator being >60 min.

### Analysis

Descriptive statistics were used to describe proportions of plans indicating coverage in each category, presence of transgender-specific exclusions, and the time to review plan websites. The *t*-tests were used to evaluate differences in indicated coverage by insurance type.

## Results

### Indication of plan coverage or exclusion

In total, 36 insurance plans were reviewed—22 commercial and 14 Medicaid. Commercial plans represented a variety of plan types: PPO (5%), EPO (22%), POS (6%), HMO (14%), Federal (6%), and Military (8%). Of these plans, commercial plans were less likely to cover puberty blockers than Medicaid plans (18% of commercial vs. 50% of Medicaid plans, *p*=0.049), but there were no differences in the other three categories of gender-affirming medical services recommended for youth (Table 1). No commercial or Medicaid plan surveyed explicitly offered coverage for all four categories.

**Table 1.** Coverage of Transition-Related Services and Transgender-Specific Exclusions by Insurance Type

Transition-related services and exclusions	All plans	Commercial plans (*n*=22), %	Medicaid plans (*n*=14), %	*p*-value^*^
Coverage of puberty blocking medications	11 (31%)	18	50	0.049^*^
Gender-affirming hormone therapy	7 (19%)	18	21	0.831
Chest reconstruction surgery	2 (6%)	0	14	0.079
Behavioral health (mental health counseling)	5 (14%)	14	14	0.978
Flagged or denied services based on gender marker	12 (33%)	32	36	
Any blanket exclusions based on diagnosis of gender dysphoria	17 (47%)	36	64	

^*^ *p*-value statistically significant if *p*<0.05.

The highest rate of coverage was for puberty blockers (31% of all plans); 19% of plans indicated covering gender-affirming hormones, 14% for mental health counseling, and only 6% for masculinizing chest surgery. Further, 33% of the plans flagged or denied coverage of services based on gender marker, and 47% of plans listed one or more trans-specific exclusions. Furthermore, the language used to describe exclusions was often inappropriate or offensive. See Table 2 for examples of exclusionary language used in SBC documents.

**Table 2.** Examples of Transgender-Specific Exclusions and Flagging or Denial Based on Gender Marker in Online Insurance Plan Information

Location on website	Transition-related services and exclusions language and examples
Transgender-specific exclusions	
Disclaimer	“Surgery, sex hormones, and related medical, psychological and psychiatric services to change a Member's sex; services and supplies arising from complications of sex transformation.”
Device policy	“Orthopedic and prosthetic devices, including penile prostheses, for the treatment of gender identity/gender dysphoria.”
Gender-marker-related restrictions	
Formulary key	Symbol for gender-limited medications
Plan booklet	“Benefits are provided for female Members for Covered Services provided by any HMO participating obstetrical/gynecological Specialist without a Referral. Covered Services include: A. Routine maternity care; B. Routine gynecological care including Pap smears; and C. Other gynecological care.”
Pharmacy policy disclaimer	“Some medications may be subject to precertification, age, gender or quantity restrictions.”
FAQs	“What are age and gender limits? Age and gender limits are restrictions on coverage of drugs designed to prevent potential harm to plan participants and promote appropriate utilization. The approval criteria are based on information from the FDA, medical literature, actively practicing consultant physicians and pharmacists, and appropriate external organizations, and are endorsed by the Pharmacy and Therapeutics Committee.”
Restrictions on male and female medication	Androgel medications fell in the male-only category. Depo-provera fell in the female-only category.
Formulary search tool requiring gender and age for medication search	“I would like to search for a drug as a: M/F, Age”
Female-specific preventive care services	Breast and cervical cancer screening, chlamydia and gonorrhea screening, domestic/interpersonal violence screening, HPV DNA testing, osteoporosis screening, well-women visits, etc.

### Accessibility of online coverage information

Length of time spent on a single review as a proxy for accessibility was recorded in 10-min increments. The median length of time to review a single plan was 50–59 min. Seven plans (19%) took >60 min to review. For commercial plans, the median review time was 50 min. The review for three commercial plans took >60 min. Medicaid plans were more varied in the time required for review, with a median review time of 50–59 min. Four (29%) Medicaid plans took >60 min to review.

## Discussion

Despite recent changes to the ACA such as Section 1557 and an increasing number of state laws requiring health insurance coverage of gender-affirming care, our study shows that rates of indication of coverage and accessibility of health insurance information for transgender and GNC youth remain poor. Fewer than half of the plans indicated coverage of any service, no plans indicated coverage of all four categories of recommended services, and nearly half of the plans indicated transgender-specific exclusions. These findings are similar to those of the National Transgender Discrimination Survey, in which 50% of transgender adults reported denial of coverage for gender-affirming surgery and 25% reported denial for hormones.^23^

In addition to poor rates of indicated coverage, accessibility of online information was suboptimal with the average plan review time >50 min for medical student research assistants who were familiar with plan information and medical language. Further, 38% had information that was classified as unclear about whether services were covered. This means that the time required for GNC youth and their families would likely be even longer, and this may lead to frustration, with many questions unanswered and possibly forgoing care as a result. One study of TY and parents showed that caregivers often used the Internet to find competent therapists who provide mental health services in their insurance plan networks.^24^ Therefore, accessibility of this type of information to consumers will be critical to achieving health equity in a population that experiences so many barriers to adequate care.

There were several limitations to our study. Information regarding actual coverage of services was not captured, and it is possible that a patient or provider could obtain different information when calling to inquire or when submitting an actual claim. The information provided online, however, is critical since this is a source many people turn to for making health care decisions.^24^ In addition, we only included insurance plans in one region in the United States, and therefore our findings may not be generalizable.

Despite these limitations, to our knowledge this is one of the first studies to describe the failure of many health insurance websites to indicate coverage of medically necessary services for TY in accordance with the ACA Section 1557. Education of insurance payors is needed to ensure appropriate coverage and accessibility of plan information for TY and their caregivers. Future research should evaluate whether these coverage barriers exist more broadly, which may in turn provide important information for policymakers and recommendations for insurance payors.

## Abbreviations Used

ACA: affordable care act
GNC: gender nonconforming
SBC: summary of benefits and coverage
TY: transgender youth
